# Mechanisms for Enhanced Hydrophobicity by Atomic-Scale Roughness

**DOI:** 10.1038/srep13790

**Published:** 2015-09-04

**Authors:** Yumi Katasho, Yunfeng Liang, Sumihiko Murata, Yasuhiro Fukunaka, Toshifumi Matsuoka, Satoru Takahashi

**Affiliations:** 1 Environment and Resource System Engineering, Kyoto University, Kyoto 615-8540, Japan; 2 Japan Oil, Gas and Metals National Corporation (JOGMEC), Chiba 261-0025, Japan

## Abstract

It is well known that the close-packed CF_3_-terminated solid surface is among the most hydrophobic surfaces in nature. Molecular dynamic simulations show that this hydrophobicity can be further enhanced by the atomic-scale roughness. Consequently, the hydrophobic gap width is enlarged to about 0.6 nm for roughened CF_3_-terminated solid surfaces. In contrast, the hydrophobic gap width does not increase too much for a rough CH_3_-terminated solid surface. We show that the CF_3_-terminated surface exists in a microscopic Cassie–Baxter state, whereas the CH_3_-terminated surface exists as a microscopic Wenzel state. This finding elucidates the underlying mechanism for the different widths of the observed hydrophobic gap. The cage structure of the water molecules (with integrated hydrogen bonds) around CH_3_ terminal assemblies on the solid surface provides an explanation for the mechanism by which the CH_3_-terminated surface is less hydrophobic than the CF_3_-terminated surface.

Interfaces between hydrophobic solids and water have attracted much attention because of their applications in a wide variety of engineering fields, including automobile windshields, building materials, green-house glass, dust-free and self-cleaning surface coatings for solar cells, sensors, anti-icing agents, ship hulls, and in the oil and gas industry^1^,^2^,^3^,^4^,^5^,^6^,^7^,^8^,^9^,^10^,^11^,^12^,^13^,^14^,^15^. The region of depleted water density at the water–hydrophobic solid surface, called the hydrophobic gap, has been studied by a number of groups^16^,^17^,^18^,^19^,^20^,^21^,^22^,^23^,^24^,^25^,^26^,^27^,^28^. Recent x-ray reflectivity studies show that the width of the hydrophobic gap on surfaces with terminal CF_3_ groups range from 1.0 Å to ~7.0 Å^22^,^23^,^26^,^27^,^28^, while for CH_3_-terminated surfaces the gap is about 1.0–4.0 Å^16^,^20^,^21^,^24^,^25^. However, molecular dynamics (MD) simulations indicate smaller hydrophobic gaps (<2.0 Å) for both CH_3_- and CF_3_-terminated surfaces^23^,^29^,^30^. One of the reasons for such a difference is thought to be the geometry of surface, or more specifically, the surface roughness. In reality, it is difficult to produce atomically and laterally flat surfaces, especially for hydrophobic surfaces, which largely rely on the fabrication of self-assembled monolayer (SAM) coatings^31^,^32^,^33^,^34^. When forming SAMs, a regular pattern might exist on the substrate. Our research motivation is to understand the effect of geometrical surface patterns on the hydrophobicity, and whether these effects explain discrepancies in the different reported hydrophobic gap values for CF_3_-terminated surfaces^22^,^23^,^26^,^27^,^28^.

To express the wettability of a surface, the contact angle *θ* is widely used^1^,^2^,^35^. It is well-known that the contact angle is affected not only by the surface chemistry but also the surface geometry^1^,^2^,^36^,^37^,^38^,^39^,^40^,^41^,^42^. The wetting behavior on rough surfaces is classified into four classes: Wenzel, Cassie–Baxter, pinning, and hemi-wicking state^1^,^2^,^36^,^37^.

In the Wenzel model, the contact angle is described by the following equation^1^,^36^,^37^
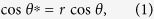
where θ^*^ is the apparent contact angle on the rough surface, *r* is the roughness factor defined by the ratio of the actual surface area to the apparent area, and *θ* is the Young contact angle. When the cosine of the apparent contact angle θ^*^ is plotted as a function of the cosine of the Young contact angle, the slope yields the roughness factor.

In the Cassie–Baxter model, the contact angle is described by the following equation^2^,^37^
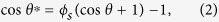
where *ϕ*_*s*_ is the ratio of solid in contact with the liquid. In this state, air is trapped between the solid and liquid. The Cassie–Baxter model works well for the large contact angle regime, while the Wenzel model works for intermediate contact angles^36^,^37^. Spori *et al.*^36^ have shown that *θ* cannot be predicted by only a roughness factor because large pinning effects are observed for the intermediate contact angle regime. By adding a term (to the Wenzel model), the pinning effect on the contact angle can be taken into account:
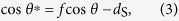
where *d*_*S*_ is the magnitude of the pinning effect (the range of *θ* is approximately from 40° to 90°). The hemi-wicking state is just a counterpart of Cassie–Baxter state of the wetting phase^36^.

MD simulations^16^,^23^,^29^,^30^,^43^,^44^,^45^,^46^,^47^,^48^,^49^,^50^ and molecular theory studies^5^,^6^ can provide insights into the structural properties of water at hydrophobic surfaces, including water–oil, water–protein, and water–solid interfaces. Water can reorganize near small apolar units, such as methane (CH_4_) and carbon tetrafluoride (CF_4_) molecules, without losing hydrogen bonds. However, an assembly of many apolar units (as they are on the solid surface) will inevitably break hydrogen bonds at the surface^5^,^6^. The key finding of this paper is that water molecules are able to form cage structures with hydrogen bonds (between water molecules within the cage) at CH_3_-terminated surfaces better than at CF_3_-terminated surfaces. This, in turn, explains why a CF_3_-terminated surface is more hydrophobic than a CH_3_-terminated surface. The strong hydrophobic nature of the CF_3_-terminated surface favors a microscopic Cassie–Baxter state at the rough surface, which enhances the observed hydrophobic gap width.

## Results

### Hydrophobic Gap

**Figure 1:**
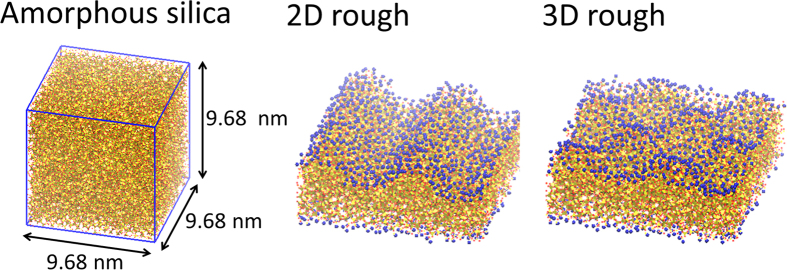
Snapshots of an amorphous silica surface, a CH_3_-terminated two-dimensional rough silica surface (n = 2), and a CH_3_-terminated three-dimensional rough silica surface (n = 2). Key: red = oxygen; yellow tetrahedron = silica tetrahedron; blue = CH_3_.

**Figure 2:**
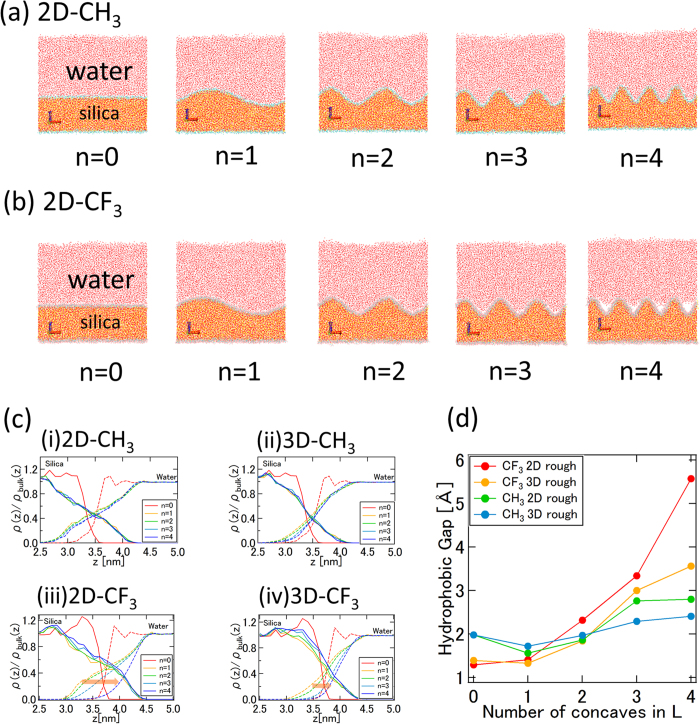
(**a**) Snapshot of the CH_3_-terminated 2D rough silica and water system at 3 ns. Key: red = oxygen; white = hydrogen; blue = CH_3_; yellow = silicon (**b**) Snapshot of the CF_3_-terminated 2D rough silica surface and water system at 3 ns. Key: blue = carbon; pink = fluoride. An enhanced hydrophobic gap was observed. (**c**) Normalized density profile of last 1 ns around the solid–liquid interface. Key: solid line = silica; dotted line = water. (i) CH_3_ 2D rough silica-water. (ii) CH_3_ 3D rough silica-water. (iii) CF_3_ 2D rough silica-water. (iv) CF_3_ 3D rough silica-water. (**d**) Hydrophobic gap width as function of number of concaves. Note: the gap is enlarged when increasing the number of concaves.

Inspired by the pioneering work of Kulinich and Farzanh^51^, we employed flat, two-dimensional (2D) and three-dimensional (3D) rough silica amorphous surfaces (Fig. 1) and investigated the interfacial water structures in contact with these surfaces. Figure 2 (a,b) show snapshots of the CH_3_- and CF_3_-terminated 2D rough silica/water system interface at 3 ns, respectively. “Vapor phase” water was observed within the concave surface regions the CF_3_-terminated 2D rough silica surface (n = 4). This implies an increase in the hydrophobic gap relative to that of the flat surface. We counted the number of water molecules within the concave regime (defined as within a distance of 0.7 nm from the average height of the substrate, i.e. the middle point of the concave) as a function of time and found the systems were equilibrated at most 1.5 ns. To evaluate changes in the hydrophobic gap, the density profile was calculated from 2.0 ns to 3.0 ns and plotted in Fig. 2(c). We found that the water boundary distance (defined as the point in the profile at which the water density falls to half of the bulk value) near the solid shifted further away with increasing the concave number (shown in panel (iii) of Fig. 2(c)). That is, the hydrophobic gap increases as the surface becomes rougher. The increase can be clearly observed in Fig. 2(d), where the hydrophobic gap is determined as the distance from the solid surface to the position which water density has half of the bulk value (see computational method for definition of solid boundary). The calculated hydrophobic gap changes from 1.3 Å for the flat case to about 5.6 Å for the CF_3_-terminated 2D rough silica surface (n = 4). It corresponds well to the various hydrophobic gap values obtained by X-ray reflectivity measurements^22^,^23^,^26^,^27^,^28^. In comparison, the hydrophobic gap values for the CH_3_-terminated surface show only a modest change over the same range of surface roughness. This also corresponds well to the consensus regarding the hydrophobic gap for the CH_3_-terminated surface^16^,^20^,^21^,^24^,^25^. In the snapshots, an enhanced hydrophobic gap was observed only in the case of water on the CF_3_-terminated 2D rough silica surface (n = 4), but from the density profiles, we can confirm that the hydrophobic gap gradually increases depending on the concave number. It indicates that the water molecules have opportunities to enter and leave the concave, as determined by a dynamic equilibrium.

The depletion layer was also calculated by Mezger *et al.* by integrating the density deficit at the solid–water interface for similar systems^23^. The method enforces the hydrophobic gap layer to have a density of 0, and therefore should be regarded as the intrinsic hydrophobic gap width. For comparison, we use the same criteria as employed in their paper. The absolute hydrophobic gaps shift to smaller values, however, the hydrophobic gap above the CF_3_-terminated 2D rough silica surface is again confirmed to gradually increase from 0.3 Å for the flat case to 2.8 Å for the n = 4 CF_3_-terminated 2D rough surface. In X-ray or neutron reflectivity experiments, the multilayer structure is assumed and only one roughness parameter is introduced in the analysis. Our results show that at least, and perhaps more than, two parameters – not only the amplitude but also the number of grooves (i.e. the roughness geometry) – can influence the hydrophobicity (or as discussed herein, the hydrophobic gap). We therefore argue that the surface geometry (among others) is responsible for the different hydrophobic gap results reported previously^22^,^23^,^26^,^27^,^28^. The mechanism of roughness enhancement of the hydrophobic gap is explained in the following sections.

### Contact Angle: Cassie–Baxter vs. Wenzel State

**Figure 3:**
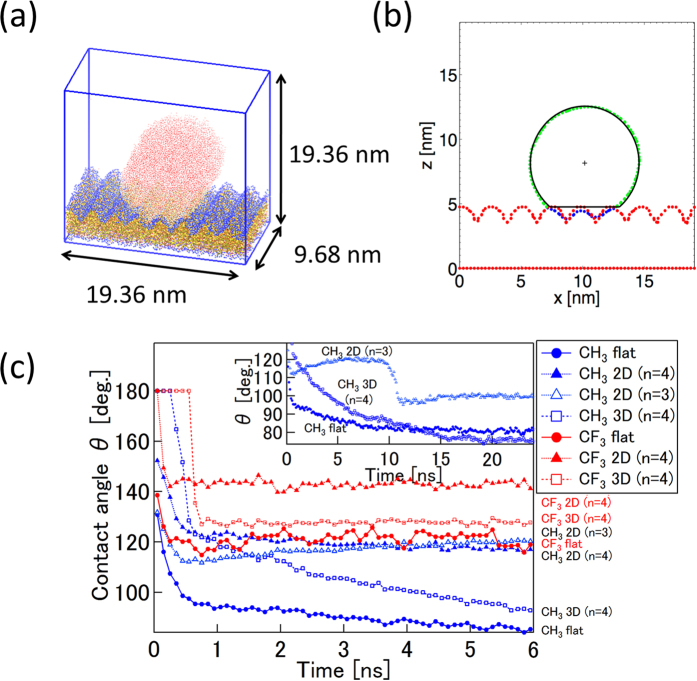
(**a**) Snapshot of the cylindrical droplet on the 2D rough silica surface at 3 ns. (**b**) The method to measure the contact angle. Key: green and blue: the point which has half of the maximum local density of water; red: the point which has 1% of the maximum local density of the substrate; blue: the point below the highest red point; black curve and line: circle fitted to the green points; black cross: center of the fitting circle. (**c**) Temporal evolution of contact angle during simulation. (CH_3_: flat, 2D rough (n = 3), 2D rough (n = 4), 3D rough (n = 4); CF_3_: flat, 2D rough (n = 4), 3D rough (n = 4)). Upper right inset shows the converged contact angles after 25 ns simulation.

We studied the wetting behavior of a cylindrical drop on a silica surface (Fig. 3). The cylindrical droplet is chosen because the contact line is straight and the contact angle is not affected by the drop size as imposed by the line tension^52^,^53^,^54^. Figure 3(c) shows the contact angle determined at 0.1 ns steps during the simulation. In the case of CF_3_-terminated silica surface, the contact angle is ~119° on the flat surface and ~141° on the 2D roughened surface, which is similar to the experimental results ranging from 111° to 133° from previous studies^22^,^32^. Our MD simulations show ranges of contact angles (including the flat, 2D, and 3D rough surfaces) in good agreement with the reported experimental data. For the CH_3_-terminated silica surface, the contact angle is ~81° on the flat surface and ~119° on 2D roughness surface. The contact angle on methylated SiO_2_ is reported as 80° to 112° in experiments^33^,^35^. Apart from Ref. 35, SAMs produced by longer molecules were used in the experiment. If SAMs produced by the same terminal molecules homogenously spread on a flat substrate, the results should be in good agreement with each other. However, the contact angle on SAMs produced by long molecules shows various values. From these results and our simulations results, we hypothesize that a portion of the SAMs produced using long molecules could exist as a lying-down structure^31^, which could play the same role of 2D roughness (as demonstrated here) and cause an increase of the contact angle^51^. The number of concaves of the 2D rough (n = 4) silica surface used in our simulations is similar to the spatial periodicity of the lying-down structure confirmed by STM in terms of the spatial period^31^. While there is a scale gap between micro and macro droplets, our simulation shows that the contact angle could be increased substantially by rough surface features that have line-patterned structure (i.e. 2D rough surface).

**Figure 4:**
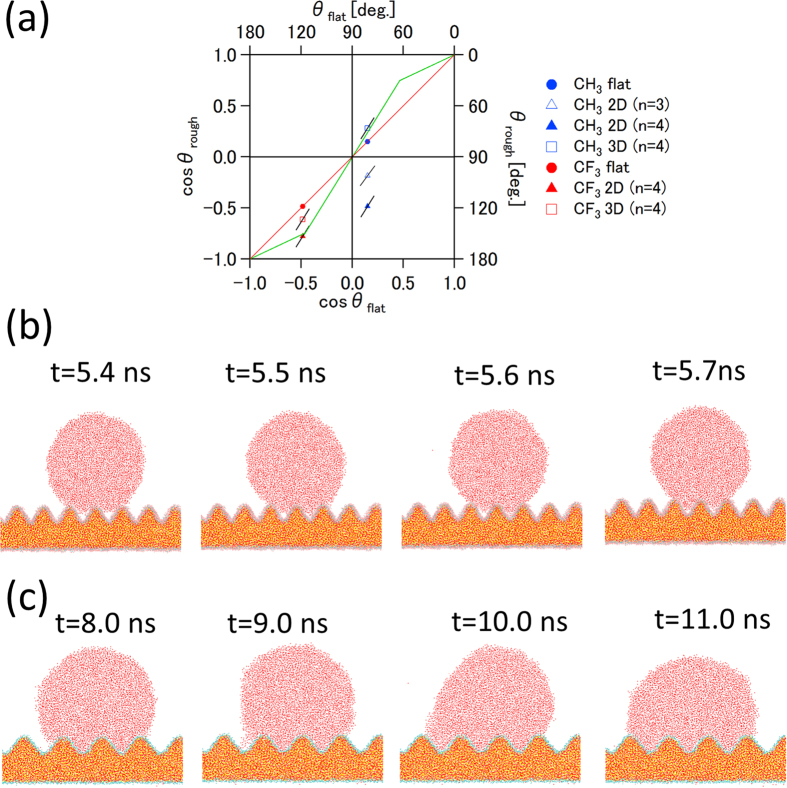
(**a**) Diagram of the effect of roughness on the contact angle. The slope of the black line is calculated by each roughness factor (i.e. the actual surface area). The green line shows the theoretical curve for Wentzel, Cassie–Baxter, and hemi-wicking models (see text for details). The red line shows theoretical curve (reference) for the droplet on a flat surface. (**b**) Snapshots of the cylindrical droplet on the CF_3_-terminated 2D rough silica surface (n = 4). The “vapor phase” below the droplet indicates that the Cassie–Baxter state was observed. (**c**) Snapshot of the cylindrical droplet on CH_3_-terminated 2D rough silica surface (n = 3). The shift of the contact angle at 9.0–10 ns as shown in Fig. 3(c) can be confirmed by the change of the position of the solid–liquid–air phase boundary.

We suggest that CF_3_-terminated silica surfaces embody a microscopic Cassie–Baxter state (Fig. 4(a)). This can be clearly seen in the snapshot (Fig. 4(b), see also Fig. 2(b)). With this hypothesis in mind, a theoretical line (green, in the range from −1.0 to around −0.5) is drawn through the data for the 2D CF_3_ case (Fig. 4(a)). The slope in this range (i.e. from −1.0 to around −0.5) corresponds to *ϕ*_*s*_ in Eq. 2, which indicates the ratios of the actual solid surfaces in contact with the liquid phase have values of 0.47 and 0.77, for the 2D and 3D rough surfaces, respectively. This slope value can be used to measure the degree of hydrophobicity. From this point of view, the 2D rough silica surface is more hydrophobic than the 3D rough silica surface in the case of CF_3_-terminated surface.

On the other hand, all cases of a droplet on CH_3_-terminated silica surfaces are in a microscopic Wenzel state (Fig. 4(a)). In the Wenzel model theory, the cosine function of the contact angle as a function of the Young contact angle is a linear function passing through the origin, and the slope of the line corresponds to the roughness factor *r* as shown in Eq. 1. The roughness factor is calculated from the surface function, namely, by the ratio of the actual surface area to the apparent area (namely, *L*_*x*_ × *L*_*y*_). For the 2D rough silica surface, the roughness factor is 1.37 and 1.59 for n = 3 and n = 4, respectively. In the case of the 3D rough silica surface (n = 4), the slope is 1.62. The green line passing through the origin (0, 0) is drawn in the range from around −0.5 to around 0.5 with a slope of 1.6 (Fig. 4(a)), which is near the roughness factor for the 2D (n = 4) and 3D (n = 4) rough silica surfaces. This theoretical line gives a predicted contact angle value for a CH_3_-terminated 3D rough silica surface of ~76°, in good agreement with the value of ~74° directly calculated from our MD simulations of the CH_3_-terminated 3D rough silica surface. To be complete, we also draw a green line from around 0.5 to 1.0 with the same slope as that in the range from −1.0 to around −0.5, as hemi-wicking state is just a counterpart of Cassie-Baxter state^36^.

The droplet contact angle for the CH_3_-terminated 2D rough silica surface is, however, not on the theoretical Wenzel line (Fig. 4(a)). Instead, *θ*_rough_ increases. This indicates that the pinning effect plays a significant role in this system, as can be seen in the snapshot in Fig. 4(c). A portion of the water molecules are attracted to the adjoining concave wall and the wetting area is enlarged. Snapshots revealed that the water droplet was pinned within the concave and depinned. The pinning and depinning process is also indicated in the inset of Fig. 3(c) by the contact angle shift around 9–10 ns for the CH_3_-terminated 2D rough surface (n = 3). Spori *et al.*^36^ demonstrated large pinning effects on square grid point pattern convexes (golf-tee shaped pillars); even with the different length scales, this pinning effect would play the same role as the 2D rough silica surface. In contrast, the 3D rough silica surface also has convexes with a square grid point pattern, but the pinning effect was not observed and wetting data followed the Wenzel model. In this case, the contact angle decreased more slowly than in the flat case in Fig. 3(c). It can be thought that the droplet is pinned, gradually depinned, and finally, reaches Wenzel state at 25 ns. We conclude that the geometry difference of the CH_3_-terminated silica surfaces causes the different equilibrium contact angles, which results from the pinning and depinning effect.

In summary, the CF_3_-terminated surface system is in microscopic Cassie–Baxter state. The CH_3_-terminated surface system is in a microscopic Wenzel state, besides the pinning effect is observed. In fact, the “vapor phase” within the concave (as mentioned above) will be thermodynamically stable if the energy of the Cassie–Baxter state is lower than that in the Wenzel state^38^. The critical contact angle (dividing the Cassie–Baxter state and the Wenzel state) can be determined by equating Eq. (1) and Eq. (2), which yields *cosθ*_*c*_ = *(Φ*_*s*_ − *1)/(r* − *Φ*_*s*_)^37^,^38^. By using the roughness factor of the rough surface in our study, and *Φ*_*s*_ = *0.47* and *0.77,* we obtained *θ*_*c*_ as 118.2° and 105.7° for 2D and 3D rough surface (n = 4), respectively. The contact angle for the CF_3_-terminated silica surface is ~119°, which is indeed larger than, and in proximity of, this critical angle.

### Droplet Anisotropy Induced by 2D Roughness

**Figure 5:**
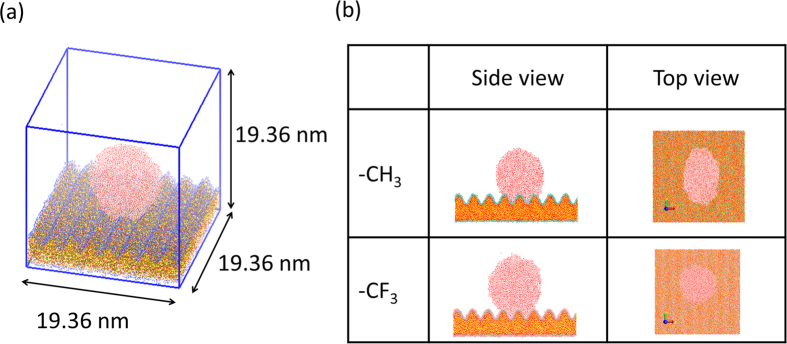
(**a**) Snapshot of the spherical droplet on the 2D rough silica surface at 3 ns. (**b**) Snapshot of the spherical droplet at 3 ns on the 2D rough CH_3_- and CF_3_-terminated silica surfaces. The anisotropic behavior of the droplet was observed in the case of the CH_3_-terminated silica surface, while the spherical shape was maintained during the simulation for the CF_3_-terminated silica surface.

Previous experiments showed that droplets in contact with a line-patterned surface elongated along the concave (micro-groove) and the contact line deviated from an ideal circle^38^. We have thus conducted simulations with spherical droplets on a 2D rough surface (Fig. 5). Figure 5(b) shows the snapshot of a spherical droplet on the 2D rough substrate at 3 ns. Interestingly, anisotropy of the droplet was observed in the CH_3_-terminated surface case, while a symmetrical droplet (with a circular contact line) was observed for the CF_3_-terminated silica. The anisotropic droplet behavior indicates that the CH_3_-terminated surface is indeed in a microscopic Wenzel state, while the CF_3_-terminated silica is in a microscopic Cassie–Baxter state. The “vapor phase” water enables the line-pattered (i.e. 2D rough) CF_3_-terminated silica surface to impart isotropic-like behavior (because the droplet sits on a cushion of vapor phase). It is reasonable that the contact angles would be different for a CH_3_-terminated silica substrate, since the effective contact lengths (i.e. contact areas if a stripe with a finite thickness is assumed) are different along the different directions. To corroborate this hypothesis, we measured the contact angles along the parallel and perpendicular directions of the 2D rough surface. The *θ*_┴_ is ~132°, whereas the *θ*_||_ is ~94°. The measured degree of wetting anisotropy, defined as the difference of the contact angle values for the two directions is about 38°. In contrast, the difference of the contact angle values for the CF_3_-terminated surface is only 6°. Here, we caution that these values should be read qualitatively as the contact angle of spherical droplet depends on the size of droplet because of the line tension effect^52^,^53^,^54^.

### Mechanisms of Superior Hydrophobicity and Microscopic Cassie–Baxter State

**Figure 6:**
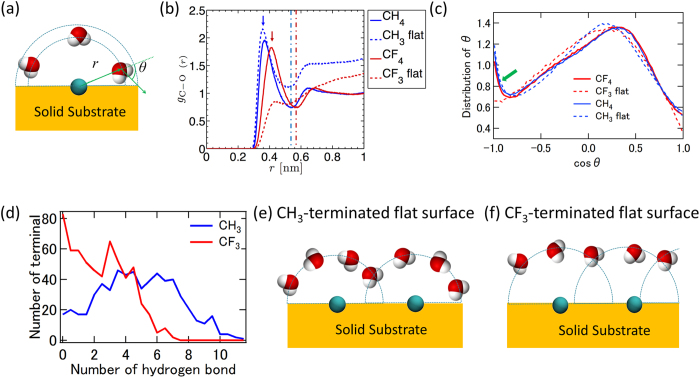
Radial, angle, and hydrogen bond distribution function of silica-water interfacial system: (a) the schematic figure of definitions of radial distance and angle (between the C…O interatomic vector and the dipole moment vector of the water molecule), (b) comparison for the water on CH_3_- and CF_3_-terminated flat surfaces and molecule-in-water system, (c) comparison for the angle distribution for C of CH_3_- and CF_3_-terminated flat surfaces and molecule-in-water system, (d) the hydrogen bond number distribution surrounding the CH_3_- and CF_3_ terminals on CH_3_- and CF_3_-terminated flat surfaces. Schematic figure of water “cage structure” for (**e**) CH_3_- and (**f**) for CF_3_-terminated flat surface. Note: the C…O radial distance cutoff is 0.54 nm and 0.57 nm for CH_3_- and CF_3_-terminated surfaces, respectively. The CF_3_-terminated surface exhibits stronger water depletion than CH_3_-terminated surface.

Using MD simulations, we have shown that the hydrophobic gap can be enhanced by roughness for a CF_3_-terminated surface. Furthermore, we found that this phenomenon is tightly connected with a microscopic Cassie–Baxter state induced by atomic-scale roughness. In particular, the CF_3_-terminated surface is more hydrophobic (with water contact angle ~119°) than a CH_3_-terminated surface (with water contact angle ~81°). To investigate the mechanism responsible for the different hydrophobicity of CH_3_- and CF_3_-terminated surfaces, the interfacial water structures were compared, including the radial distribution function (RDF) between C atoms of CH_3_/CF_3_ unit and O atoms of water, the angle distribution function (ADF) between the C…O interatomic vector and the dipole moment vector of the water molecule, and the hydrogen bond number distribution (between water molecules) surrounding each individual CH_3_ and CF_3_ unit. These values were calculated in each case by using the trajectory from 2.0 to 3.0 ns (as shown in Fig. 2). The schematic definition of RDF and ADF are shown in Fig. 6(a). Calculated RDFs of CH_3_- and CF_3_-terminated silica systems are shown in Fig. 6(b). To separate the influence of geometry in the RDF and ADF calculations, two additional simulations were performed on systems with bulk water containing only one CH_4_ or CF_4_ molecule.

All RDFs of the CH_3_-terminated surface and CH_4_ molecule-in-water systems have a peak at 0.36 nm, while all RDFs of CF_3_ terminal surfaces and CF_4_ have a peak at 0.42 nm. This size difference leads to different hydrophobicities on the flat surfaces and a completely different microscopic wetting state for rough surfaces. As shown in Fig. 6(b), the water has a clear “cage structure” surrounding the CH_4_ and CF_4_ molecules. The structure is maintained to a great extent for the CH_3_-terminated surface, as evidenced by the similar peak height, however, the structure is seriously disturbed for CF_3_-terminated surface, as evidenced by the less featured peak in the RDF, presumably because of the large size of the CF_3_ unit (that is, less spare space on the surface). The first minimum in the RDF is 0.54 and 0.57 nm for the CH_4_ and CF_4_ molecules, respectively. These values were used as the cutoff distance for the calculation of ADFs. As shown in Fig. 6(c), the angle of the water dipole presents two peaks for both CH_4_ and CF_4_ molecule-in-water systems, which indicates a preferred orientation of water molecules within the cage (like methane clathrate hydrate)^5^,^6^,^49^,^50^. The peak at cos *θ* = −1.0 is almost diminished for CF_3_-terminated surface, while it is essentially the same for the CH_3_-terminated surface and CH_4_ molecule-in-water systems. The water coordination number of CX_4_ molecule is roughly 23.6 for CF_4_ and 20.4 for CH_4_, respectively. It is reduced more than half to a range with a maximum value 8.0 for CF_3_ terminals (the average is 4.0) and to a less extent 12.0 for CH_3_ terminals (the average is 6.4) on the surface. Remarkably, the hydrogen bond number (surrounding an individual CH_3_ unit) on the CH_3_-terminated surface is much higher that on the CF_3_-terminated surface (Fig. 6(d)). This indicates that a better water “cage structure” has been formed surrounding CH_3_ terminals than CF_3_ terminals. Furthermore, it explains why the CH_3_-terminated surface is less hydrophobic than the CF_3_-terminated surface, which, in turn, leads a microscopic Cassie–Baxter state for the CF_3_-terminated rough surface (as discussed above). A simple schematic explanation (for better water “cage structure”) would be that CH_3_ group is smaller than CF_3_ group (Fig. 6 (e,f)). In a previous study, it has been shown that water experiences a weaker van der Waals interaction and stronger depletion from CF_3_ terminated surface than from CH_3_ terminated surface^48^. We believe that the poorer water “cage structure” (surrounding CF_3_ terminals than CH_3_ terminals) provides the underlining mechanisms for weaker van der Waals interactions.

**Figure 7:**
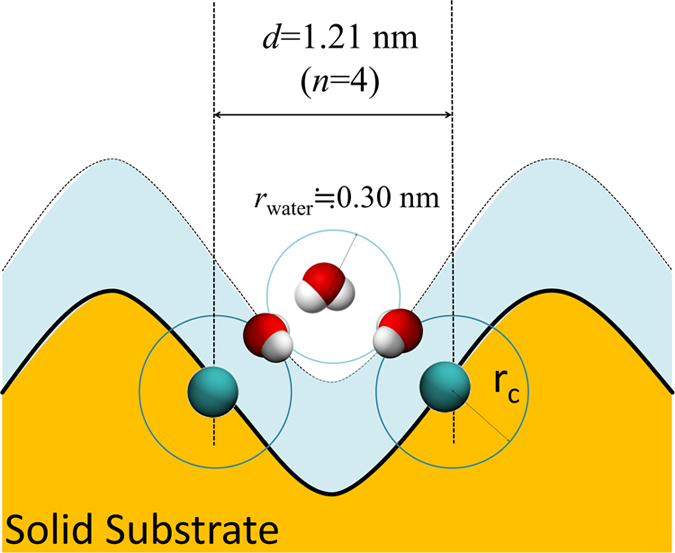
Schematic figure illustrating the mechanism for enhancement of hydrophobic gap by roughness for CF_3_-terminated silica surface.

As mentioned above, the calculated ratio of the actual solid surface (of 2D CF_3_-terminated rough surface) in contact with the liquid water phase is about 47%. This means almost half of the solid is in contact with the “vapor phase” indicating that water molecules do not exceed the middle points of the rough surface. The distance between the middle points of adjoining convexes *d* is 1.21 nm in the case of the 2D rough surface with n = 4; the RDF between the oxygens of water molecules has a peak at 0.28 nm^55^; and the distance between C of CF_3_ and O of water is about 0.42 nm. This means that roughly three water molecules are in line with the middle points of the concave with n = 4 (Fig. 7). The isolated water is not energetically favorable; instead, hydrogen bonding between water molecules is the key factor that allows liquid water on a CF_3_-terminated rough surface to be in a microscopic Cassie–Baxter state.

## Discussion

We investigated the enhanced hydrophobic gap using 2D or 3D rough, and CH_3_- or CF_3_-terminated, silica surfaces. The hydrophobic enhancement was especially significant for the 2D rough CF_3_-terminated silica, which has the narrowest concaves. The observed differences of the hydrophobic gap are thus explained by substrates bearing slightly different surface geometries^22^,^23^,^26^,^27^,^28^. Furthermore, we studied the effect of roughness to the contact angle. The CH_3_-terminated surface was found to exist in a microscopic Wenzel state and pinning–depinning behavior was observed. The CF_3_-terminated silica surface presented in a microscopic Cassie–Baxter state. The RDF calculation results show us that a CF_3_ group is significantly larger than a CH_3_ group, which disturbs the water “cage structures” at water–solid interface surrounding the apolar unit. This causes a much-enhanced hydrophobicity and an enlarged hydrophobic gap on the rough surface with a microscopic Cassie–Baxter state. On the other hand, the CH_3_-terminated surface only presents a marginally enhanced hydrophobic gap and Wenzel state on the CH_3_ 2D rough silica surface. Our study helps rationalize why CF_3_-terminated surfaces are in general very hydrophobic, for which the atomic-roughness (of SAM) could have already played a significant role.

It has been suggested that interfacial water against a hydrophobic solid surface can have a structure similar to that of the liquid–vapor interface. Recent vibrational sum frequency spectroscopy studies have shown, however, that water molecules at CH_3_ terminated silica surface have an ordered, ice-like structure^9^. In addition, it presents a different vibrational coupling for isotope dilution experiments when compared with the liquid–vapor interface^34^. The water “cage structure” surrounding the CH_3_ unit can thus be responsible for the ice-like structure^9^ and the observed vibrational coupling difference^34^. Furthermore, it has recently been reported that the fluorinated (i.e., CF_3_-terminated) surface exhibits both hydrophobic and oleophobic character^10^,^11^,^12^,^13^,^14^,^15^, where surface roughness is believed to play a significant role. Our study highlights the importance of considering the atomic-scale surface roughness in combination with the surface chemistry.

## Computational Methods

### Structure of the Rough Silica Surface

For simplicity, we assumed a surface function as follows:
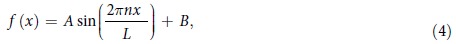
where *A* is the amplitude of the surface function, *B* is the average of the surface function, *n* is the number of concaves within *L*, and *L* is the length of the simulation box in the *x*-direction. We refer to this surface as the 2D rough surface. We proposed another surface function as follows:
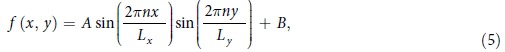
where *L*_*x*_ and *L*_*y*_ are the length of the simulation box in the *x*- and *y*-directions, respectively. We call this the 3D rough surface. In the case of the 2D rough surface, the rms roughness becomes 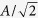, and for the 3D rough surface, the rms roughness is *A*/2. In this simulation, we assumed *A* = 7.07 Å for all calculations. When *A* = 7.07 Å, the rms roughness was 5.00 and 3.53 Å for the 2D and 3D rough surfaces, respectively, which correlate with the same roughness magnitude as in experiment^31^.

A large well-relaxed model of vitreous silica^56^ was used in this study. It is a cubic cell composed of 20000 silicon atoms and 40000 oxygen atoms with a cell length of 9.68 nm. We prepared the 2D and 3D rough silica surfaces (Fig. 1) by cutting the silica glass with the number of concaves from 0 to 4. The average thickness of the obtained substrate is 3.0 nm. CH_3_ or CF_3_ groups were attached to unbonded terminals of both the upper and bottom surfaces. The percentages of single Si-CX_3_ is ~70–80% and the rest are germinal Si-(CX_3_)_2_.

### Details of Molecular Dynamics Simulations

All MD simulations were performed using the GROMACS package^57^. The silica glass, CH_3_ termini, and CF_3_ termini were described by the CLAYFF^58^, GROMOS54A7^59^,^60^, and OPLS^61^ potential force fields, respectively. When the terminal CX_3_ unit was attached to an unbonded Si, its charge was adjusted according to the number of termini to keep the local charge neutral. We used the Si-C bond length proposed by Sun^61^, with a bond stretching constant as implemented in GROMOS54A7^59^,^60^. A nominal C-Si-C angle bending parameter (251.208 kJ/mol) was used when it is germinal Si-(CX_3_)_2_. The CH_3_ and CF_3_ groups were found to be re-distributed due to their repulsive forces between CX_3_ groups. The nearest inter-molecule C-C distances were 0.35 and 0.40 nm for CH_3_- and CF_3_-terminated surfaces, respectively. That is, the larger size of CF_3_ terminals^62^,^63^ were reproduced well in our surface models. The water molecules were modeled by an extended simple point charge model (SPC/E)^64^.

The simulations were performed at a constant temperature of 300 K using the Berendesen thermostat^65^. The total simulation times were 3.0 ns for the interface system and 6.0 ns for the droplet system for contact angle calculation. If the contact angle was not converged, an additional 19 ns simulation was conducted after this initial 6 ns run. Particle Mesh Ewald summation^66^ was used for the electrostatic interactions, and a cutoff of 11 Å was used for the van der Waals interactions. A 1.0 fs time step was used and the coordinates output every 1.0 ps.

### Interface System and Hydrophobic Gap

For the hydrophobic gap calculations, all of the prepared rough silica substrates were used (CH_3_ or CF_3_ and 2D or 3D rough and 0 ≤ n ≤ 4). 16000 water molecules (initially 9.68 nm × 9.68 nm × 5.12 nm cubic water group) were used to construct the interface system. We defined the width of the hydrophobic gap to account for the shift amount of the water boundary on the rough side of the silica. The bottom of the solid surface was defined as zero position (*z* = *0*) of the density profile. The intersection position of the averaged density profiles of rough solid cases (1 ≤ n ≤ 4) and flat solid density profile was determined as the solid boundary. The water boundary (on the side of solid surface) was determined by the position which water density profile has half of the bulk density. The hydrophobic gap was defined as the difference in the position of the solid boundary and water boundary. The rough solid density profile basically follows the same arcsine function. Therefore, the averaged density can be used. However, the intersection position of the 2D rough surface and flat surface system is slightly different from the intersection position for the 3D case. Therefore, a minor difference of the hydrophobic gap width is anticipated for the flat case when the hydrophobic gap as function of concave number is shown (as shown in Fig. 2(d)).

### Cylindrical Droplet and Contact Angle

To evaluate the contact angle, a cylindrical droplet was chosen considering that the cylindrical droplets are not affected by line tension since the contact line in the *y*-direction is straight^52^,^53^. Also, it is easy to determine the contact angle in both aspects of fitting method and computational cost (as the size along the *y*-direction can be small)^54^. For the cylindrical droplet simulation, the silica substrates were used which have either no concave (flat surface) or 4 concaves within the 9.68 nm length. The size of the substrate was doubled along the *x-*direction and 19452 water molecules (initially, 7.70 nm × 7.70 nm × 9.68 nm) were set above the substrate (Fig. 3(a)).

The density map was obtained every 0.1 ns. In this method, the water boundary was determined by the position which has half of the maximum local density of liquid water (roughly speaking, the bulk density of liquid water). The solid boundary was determined by the uppermost position that has 1% of the maximum local density of the solid. The contact angles were calculated every 0.1 ns by least-square fitting to a circle with water boundary points above the solid boundary (Fig. 3(b)).

To confirm the pinning effect in the case of the droplet on CH_3_-terminated 2D rough surface, an additional simulation using a CH_3_-terminated silica surface which has 3 concaves in 9.68 nm was conducted.

### Spherical Droplet

For a spherical droplet, the 2D rough silica substrates which have no concave (flat surface) or 4 concaves were used. The substrate was doubled in both *x*- and *y-* directions. 11417 water molecules (roughly, 7.0 nm × 7.0 nm × 7.0 nm) were set above the substrate (Fig. 5(a)). 3 ns simulations were conducted for both droplet simulations on the CH_3_- and CF_3_-terminated silica.

## Additional Information

**How to cite this article**: Katasho, Y. *et al.* Mechanisms for Enhanced Hydrophobicity by Atomic-Scale Roughness. *Sci. Rep.* **5**, 13790; doi: 10.1038/srep13790 (2015).
